# Study of key amino acid residues of GH66 dextranase for producing high-degree polymerized isomaltooligosaccharides and improving of thermostability

**DOI:** 10.3389/fbioe.2022.961776

**Published:** 2022-08-10

**Authors:** Qianru Lin, Huanyu Wang, Yingying Xu, Dongxue Dong, Qingzhen Miao, Jing Lu, Mingsheng Lyu, Shujun Wang

**Affiliations:** ^1^ Jiangsu Key Laboratory of Marine Bioresources and Environment/Jiangsu Key Laboratory of Marine Biotechnology, Jiangsu Ocean University, Lianyungang, China; ^2^ Co-Innovation Center of Jiangsu Marine Bio-industry Technology, Jiangsu Ocean University, Lianyungang, China

**Keywords:** dextranase, site-directed mutation, high-degree polymerization, isomaltooligosaccharide, molecular docking

## Abstract

Obtaining high-degree polymerized isomaltose is more difficult while achieving better prebiotic effects. We investigated the mutation specificity and enzymatic properties of SP5-Badex, a dextranase from the GH66 family of *Bacillus aquimaris* SP5, and determined its mutation sites through molecular docking to obtain five mutants, namely E454K, E454G, Y539F, N369F, and Y153N. Among them, Y539F and Y153N exhibited no enzymatic activity, but their hydrolysates included isomaltotetraose (IMO4). The enzymatic activity of E454G was 1.96 U/ml, which was 3.08 times higher than that before mutation. Moreover, 70% of the enzymatic activity could be retained after holding at 45°C for 180 min, which was 40% higher than that of SP5-Badex. Furthermore, its IMO4 content was 5.62% higher than that of SP5-Badex after hydrolysis at 30°C for 180 min. To investigate the effect of different amino acids on the same mutation site, saturation mutation was induced at site Y153, and the results showed that the enzyme activity of Y153W could be increased by 2 times, and some of the enzyme activity could still be retained at 50°C. Moreover, the enzyme activity increased by 50% compared with that of SP5-Badex after holding at 45°C for 180 min, and the IMO4 content of Y153W was approximately 64.97% after hydrolysis at 30°C for 180 min, which increased by approximately 12.47% compared with that of SP5-Badex. This site is hypothesized to rigidly bind to nonpolar (hydrophobic) amino acids to improve the stability of the protein structure, which in turn improves the thermal stability and simultaneously increases the IMO4 yield.

## Introduction

Dextranase is a hydrolase capable of specifically cleaving the α-1,6 glycosidic bond of dextran (Suzuki et al., 2012) and plays a major role in food and medical industry (Khalikova et al., 2005). Dextranases have different species. Based on amino acid sequence homology, glucose dextranases (EC3.2.1.70) are classified into glycosidic hydrolase families 13 and 15, isomaltose dextranases (EC3.2.1.94) and isomaltotriose dextranases (EC3.2.1.95) are classified into glycosidic hydrolase families 27 and 49, respectively, and endo-dextro glycosidases are classified into glycosidic hydrolase families 49 and 66. No homologous sequences existed between the two families 49 and 66. Dextroglycosidases can be obtained through various sources. *Arthrobacter* spp. is the main bacterial source of GH49 family dextroglycosidases and *Streptococcus* spp*.* is for GH66 family dextroglycosidases. We here investigated the marine bacterium *Bacillus aquimaris* SP5, which is the bacterial source of GH66 family dextroglycosidases.

According to the analysis of protein sequences of known dextranases performed using comparative databases, the gene and protein sequences of dextranases vary greatly in size. ClustalW (http://www.ebi.ac.uk/clustalw/) includes many known protein sequences as comparative sequences. The phylogenetic tree demonstrated that great differences exist in the structures of different dextranases. Wild-type dextranases are usually unstable and exhibit slightly lower enzyme activity and product turnover rates. Through cloning and expression, engineered strains producing enzymes with improved stability and functional activity can be obtained. Kim et al. (2009) co-constructed a dextro-anhydrase gene of *Arthrobacter oxydans* origin and an α-amylase gene of *Klebsiella pneumoniae* origin into *Escherichia coli* and successfully produced an enzyme with bifunctional activity. Park et al. (2012) recombinantly expressed *Thermoanaerobacter pseudethanolicus*-derived dextroglycosidase in *E. coli* with an optimum reaction temperature of 76°C.

Compared with chemical catalysts, biocatalysts are more efficient and stable, can perform the catalytic task better, and reduce plant costs, and are also greener, so dextranase with higher enzyme activity and better stability has an invaluable role in industry, food and pharmaceutical industries (Espina et al., 2021). Also, having good thermal stability is the basis for the enzyme to be stable in the sugar industry and other industries (Liu et al., 2021). In general, dextranases with higher enzyme activity and stability are invaluable for different industries, including food and pharmaceutical industries. Dextranase is used in cancer treatment as a biospecific antigen, such that cancer-inhibiting drugs act only on cancer cells, thereby protecting healthy human cells from damage due to drug. L-glucuronidase can effectively inhibit plaque production in humans and maintain oral health (Ryu et al., 2000). In the sugar industry, it can inhibit dextran production and improve the yield of sucrose (Borji et al., 2019). Meanwhile, the hydrolysis product of the dextranase isomalt polysaccharide can also promote the growth of intestinal probiotics, promote intestinal peristalsis, reduce the occurrence of intestinal diseases, and promote intestinal health (Kubik et al., 2004; Goffin et al., 2011). Dextran is typically not digested and degraded in the small intestine because of the lack of relevant hydrolases; however, in the large intestine, it is easily digested due to the presence of probiotics such as *Lactobacillus* sp. and *Bifidobacterium* sp. (Olano-Martin et al., 2000; Sarbini et al., 2011). Some researchers have isolated various dextranase-producing bacteria from human feces. These microorganisms and their enzymes may be better adapted to the human intestinal environment and may offer some guidance for the application of dextranase in humans (Kim et al., 2015). Taisuke et al. investigated dextro-glycosaminidase-based therapies (Tiryaki et al., 2020), demonstrating the potential of dextranase in the development of intestinal prebiotics. Huang et al. (2020) effectively biosynthesized isomaltooligosaccharides (IMOs) with reduced by-product glucan production. Gan et al. (2014) used dextransucrase and dextranase to synergistically catalyze oligodeoxyglucose production from sucrose. Bertrand et al. (Bertrand et al., 2014) and Chalane et al. (2017) prepared immobilized enzyme reactors capable of continuously producing IMOs with a controlled molecular weight and high stability of enzyme activity by using epoxy resin-immobilized dextrose. Tingirikari et al. (2017) co-immobilized dextran sucrase and dextranase with alginate microspheres and then used them to convert sucrose to IMOs in orange juice to produce orange juice as a functional health drink.

The environment inside and outside the cell varies greatly and is more unstable, such as more acidic or alkaline hydrolysis environments or more complex substrates, so it is also more urgent to make the enzyme still work stably in the industrial environment (Littlechild, 2015). In recent years, protein engineering is emerging as a burgeoning tool to improve the enzymatic activity and thermal stability of wild enzymes (Chen et al., 2022). And strains that meet research needs can be obtained through genetic modification with targeted mutations or truncations. Moreover, predictive analysis can be performed through molecular docking, a common tool in modern molecular biology, to select mutation sites. Molecular docking refers to the computerized placement of ligand molecules in the binding pocket (active site) of protein receptors. This is followed by the evaluation of the goodness of the interaction between ligand molecules and protein receptors according to the principles of energy complementarity, geometric complementarity, and chemical environment complementarity, thus predicting the binding force and conformation between ligands and receptors and obtaining their optimal binding mode (Huang and Zou, 2010). In molecular docking, molecules are bound by interaction forces, such as electrostatic, hydrogen bonding, and hydrophobic (Shoichet et al., 1999; Brenk et al., 2006). Residues located in the catalytic module of Man1E and the CBM of Man1E were identified by Liu et al. (2020a). Through superposition analysis and molecular docking. The recombinant enzyme had a significantly increased thermal stability at 60°C (Liu et al., 2020b).

In this study, mutation sites were identified by molecular docking, mutation analysis was performed on the sites, and one site was selected for saturation mutation. This mutation finally led to an improvement in the enzymatic activity and thermal stability of dextranase SP5-Badex and thus production of a higher content of highly polymerized isomaltose.

## Materials and methods

### Strains and reagents

*Bacillus aquimaris* SP5 cells were maintained in our laboratory. The plasmid Pet29a was purchased from Hangzhou Baosai Biotechnology Company. Receptor cells *E. coli* DH5α and *E. coli* BL21 (DE3) were provided by Shanghai Tiangen Biochemical Technology (China). LBK medium is LB medium containing 50 μg/ml kanamycin, and the final concentration of Isopropyl-beta-D-thiogalactopyranoside (IPTG was 0.1 mM.

### Construction of mutant vector

Badex was modeled in 3D at NCBI and observed using Pymol. The Badex protein model was molecularly docked with isomaltotetraose (IMO4), isomaltopentaose (IMO5), and isomaltohexaose (IMO6) by using AutoDuck Vina, and the amino acid sites with the best binding and hydrogen bonding abilities were selected for mutation. Primers were designed and sent to Shanghai Biotechnology for synthesis. The DNA fragment of *B. aquimaris SP5* was 1722 bp. E454K was amplified using gene-specific primers (F 5′-CGC​GAT​GGG​GTG​GAG​AAA​ACG​GAA​ACA​GAA​G-3′ and R 5′-TCT​CCA​CCC​CAT​CGC​GGA​GTA​GAT​TC-3′). E454G was amplified using gene-specific primers (F 5′-CGC​GAT​GGG​GTG​GAG​GAA​ACG​GAA​ACA​GAA​G-3′ and R 5′-CCT​CCA​CCC​CAT​CGC​GGA​GTA​GAT​TC-3′). Y539F was amplified using gene-specific primers (F 5′-CAA​CAC​CTG​ATG​CCT​TTC​AGG​GTT​CAC​CTC​T-3′ and R 5′-AAG​GCA​TCA​GGT​GTT​GCC​ACC​CAG​AC-3′). N369F was amplified using gene-specific primers (F 5′GTA​AAG​GCA​AGC​TGA​TTA​CGG​TCC​TGG​CTG​C-3′ and R 5′-ATC​AGC​TTG​CCT​TTA​CTG​TAT​TTA​CT-3′). Y153N was amplified using gene-specific primers (F 5′AAT​GGG​CTG​CAA​TTT​AAC​GAC​TGG​CAG​T-3′ and R 5′-TAA​ATT​GCA​GCC​CAT​TGA​TGT​GGA​AT-3′).

### Site-directed mutagenesis of *B. aquimaris* SP5

The SP5 plasmid was used as the DNA template for PCR mutation. The PCR amplification system is shown in Table 1, and the PCR amplification conditions are presented in Table 2. The PCR products of positive clones were selected. Then, 5 µl of the PCR amplification products were cloned into 50 μl *E. coli* DH5α receptor cells. The cells were first placed on ice bath for 30 min, then in a water bath at 42°C for 2 min, and immediately placed again smoothly on ice bath for 2 min. Then, 900 μl LB medium were added to the cells, mixed well, and placed on a shaker at 37°C, 150 rpm for 1 h. Again, 900 μl of LB medium were added to the mixture, mixed well, and incubated on a shaker for 1 h at 37°C, 150 rpm. This would allow the receptor cells mark the mutated gene. Then, 150 μl of bacterial solution was applied to LBK solid medium. After complete absorption of the solution, the plate was inverted and incubated in a constant temperature incubator at 37°C for 12–16 h. A single colony was picked from the plate, inoculated into LBK liquid medium, and incubated on a shaker at 37°C, 180 rpm for 12 h. The plasmids were extracted using a centrifugal column plasmid extraction kit (Shanghai Tiangen Biochemical Technology, China). The extraction results were verified through 1% agarose gel electrophoresis.

**TABLE 1:** PCR amplification system.

Ingredients	50 μl system	Final concentration
DNA Template	100 ng	—
Forward mutation primers	2 μl	400 nM
Reverse mutation primers	2 μl	400 nM
5 ×Fast alteration buffer	10 μl	1×
Fast alteration DNA polymerase (2.5 U/ml)	1.5 μl	0.075 U/μl
RNase-Free dd H_2_O	Up to 50 μl	—

**TABLE 2:** PCR amplification conditions.

Content	Temperature/(°C)	Time	Circulation
Pre-mutability	95	2 min	1×
Mutability	94	20 s	18×
Annealing	55	10 s	
Extension	68	2.5 min	
Additional extensions	68	5 min	1×

### Saturation mutations of *B. aquimaris* Y153

The saturation mutation technique facilitates generation of a library of possible amino acid variants at the same locus through random codons (Katano et al., 2017), while achieving combinations of beneficial amino acids (Fernandez-Lopez et al., 2016). Saturation mutations were performed on locus Y153, and saturation mutation primers were designed as listed in Table 3.

**TABLE 3:** Primer design for Y153 saturation mutation.

Primer	5–3′
Y153H-F	AAT​GGG​CTG​CAA​TTT​CAC​GAC​TGG​CAG​T
Y153H-R	GAA​ATT​GCA​GCC​CAT​TGA​TGT​GGA​AT
Y153R-F	CAT​CAA​TGG​GCT​GCA​ATT​TCG​CGA​CTG​GCA​GTA​CAA​A
Y153-R	TTT​GTA​CTG​CCA​GTC​GCG​AAA​TTG​CAG​CCC​ATT​GAT​G
Y153K-F	CAT​CAA​TGG​GCT​GCA​ATT​TAA​AGA​CTG​GCA​GTA​CAA​A
Y153K-R	TTT​GTA​CTG​CCA​GTC​TTT​AAA​TTG​CAG​CCC​ATT​GAT​G
Y153I-F	CAT​CAA​TGG​GCT​GCA​ATT​TAT​CGA​CTG​GCA​GTA​CAA​A
Y153I-R	TTT​GTA​CTG​CCA​GTC​GAT​AAA​TTG​CAG​CCC​ATT​GAT​G
Y153L-F	CAT​CAA​TGG​GCT​GCA​ATT​TCU​CGA​CTG​GCA​GTA​CAA​A
Y153L-R	TTT​GTA​CTG​CCA​GTC​GAG​AAA​TTG​CAG​CCC​ATT​GAT​G
Y153W-F	CAC​ATC​AAT​GGG​CTG​CAA​TTT​UGG​GAC​TGG​CAG​TAC​AAA​C
Y153W-R	GTT​TGT​ACT​GCC​AGT​CCC​AAA​ATT​GCA​GCC​CAT​TGA​TGT​G
Y153A-F	CAT​CAA​TGG​GCT​GCA​ATT​TGC​CGA​CTG​GCA​GTA​CAA​A
Y153A-R	TTT​GTA​CTG​CCA​GTC​GGC​AAA​TTG​CAG​CCC​ATT​GAT​G
Y153M-F	CAC​ATC​AAT​GGG​CTG​CAA​TTT​AUG​GAC​TGG​CAG​TAC​AAA​C
Y153M-R	GTT​TGT​ACT​GCC​AGT​CCA​TAA​ATT​GCA​GCC​CAT​TGA​TGT​G
Y153P-F	CAT​CAA​TGG​GCT​GCA​ATT​TCC​CGA​CTG​GCA​GTA​CAA​A
Y153P-R	TTT​GTA​CTG​CCA​GTC​GGG​AAA​TTG​CAG​CCC​ATT​GAT​G
Y153C-F	AAT​GGG​CTG​CAA​TTT​TGC​GAC​TGG​CAG​T
Y153C-R	CAA​AAT​TGC​AGC​CCA​TTG​ATG​TGG​AAT
Y153F-F	CAT​CAA​TGG​GCT​GCA​ATT​TTT​TGA​CTG​GCA​GTA​CAA​A
Y153F-R	TTT​GTA​CTG​CCA​GTC​AAA​AAA​TTG​CAG​CCC​ATT​GAT​G
Y153V-F	CAT​CAA​TGG​GCT​GCA​ATT​TGT​CGA​CTG​GCA​GTA​CAA​A
Y153V-R	TTT​GTA​CTG​CCA​GTC​GAC​AAA​TTG​CAG​CCC​ATT​GAT​G
Y153G-F	CAT​CAA​TGG​GCT​GCA​ATT​TGG​CGA​CTG​GCA​GTA​CAA​A
Y153G-R	TTT​GTA​CTG​CCA​GTC​GCC​AAA​TTG​CAG​CCC​ATT​GAT​G
Y153S-F	AAT​GGG​CTG​CAA​TTT​TCC​GAC​TGG​CAG​T
Y153S-R	GAA​AAT​TGC​AGC​CCA​TTG​ATG​TGG​AAT
Y153Q-F	CAT​CAA​TGG​GCT​GCA​ATT​TCA​GGA​CTG​GCA​GTA​CAA​A
Y153Q-R	TTT​GTA​CTG​CCA​GTC​CTG​AAA​TTG​CAG​CCC​ATT​GAT​G
Y153D-F	AAT​GGG​CTG​CAA​TTT​GAC​GAC​TGG​CAG​T
Y153D-R	CAA​ATT​GCA​GCC​CAT​TGA​TGT​GGA​AT
Y153E-F	CAT​CAA​TGG​GCT​GCA​ATT​TGA​AGA​CTG​GCA​GTA​CAA​A
Y153E-R	TTT​GTA​CTG​CCA​GTC​TTC​AAA​TTG​CAG​CCC​ATT​GAT​G
Y153T-F	CAT​CAA​TGG​GCT​GCA​ATT​TAC​CGA​CTG​GCA​GTA​CAA​A
Y153T-R	TTT​GTA​CTG​CCA​GTC​GGT​AAA​TTG​CAG​CCC​ATT​GAT​G

### Induced expression of mutant enzymes

*E. coli* DH5α plasmids (5 μl) were incorporated into *E. coli* BL21 (DE3) expression cells. The cells were placed on ice bath for 30 min, in a water bath at 42°C for 1–2 min, and then immediately and smoothly on ice bath for 2–3 min. Then, 900 μl of LB medium were added to these cells and mixed well. The cell suspension was then placed on a shaker at 37°C, 150 rpm for 1 h, then, the strain was spread on plates and incubated at 37°C for 12–16 h. A single colony was selected and inoculated on the LBK solid medium.

A single colony was picked from the plate medium and inoculated in 20 ml LBK liquid medium on a shaker at 37°C, 180 rpm for 4 h for activation culture. The cells were allowed to grow to the log phase. Then, an absorbance of approximately 0.6 at OD_600_ nm was set for the cell suspension. Subsequently, the bacterial solution (2 ml) was added to 50 ml LBK liquid medium for fermentation for 5–6 h until the OD_600_ nm value of the bacterial solution increased to approximately 0.8. Then, IPTG was added to the bacterial solution and incubated on a shaker at 16°C, 180 rpm for 24 h.

The bacterial solution was centrifuged at 8,000 rpm for 15 min, the supernatant was discarded, and the precipitate was mixed with 10 ml of 0.1 M PBS buffer by blowing. The cell suspension was recentrifuged at 8,000 rpm for 10 min, the supernatant was discarded, and the precipitate was mixed with 10 ml of 0.1 M pH 7.4 PBS buffer by blowing and washed. Then, the cell precipitate was mixed with 5 ml of 0.1 M pH 7.4 PBS buffer, and an ultrasonic crusher was used to crush the cells for 12 min. The crushed cells were centrifuged at 8,000 rpm for 10 min, and the supernatant was collected and used as the crude enzyme solution.

### Protein expression and purification

After the crude enzyme solution was filtered through a 0.22-μm membrane, the mutant enzyme was purified using magnetic beads and eluted with 20, 40, 60, 80, 100, 200, and 300 mM imidazole. The eluate and effluent were then collected. The protein concentration was measured using the BCA protein concentration kit. The eluate with lower protein content was concentrated using an ultrafiltration tube and the concentrate was collected. A 10% polyacrylamide gel was prepared, and the eluate was sampled at 3 mg protein content at 80 V for 30 min. The voltage was adjusted to 120 V until the end of gel running. The gel was stained with Coomassie Brilliant Blue and decolorized with decolorizing solution overnight.

### Enzyme assay and enzymatic properties of SP5 and its variants

To test the dextranase activity by using the 3,5-Dinitrosalicylic acid (DNS) method, 150 μl of 3% dextran T70 solution was added to the test tube and placed in a water bath for 2–3 min. Then, 50 μl of mutant was added to react for 15 min; the process should be strictly controlled. Subsequently, 200 μl of DNS was added to terminate the reaction, and the mixture was boiled for 5 min to develop color. Pure water (3 ml) was added to the test tube and mixed well. Then, 200 μl of the mixture was pipetted into a 96-well plate, absorbance at OD_540_ nm was measured, and enzyme activity was calculated. The optimum enzyme activity of the mutant enzyme at 30–50°C, its temperature stability at 30–50°C pH 7.5 and its optimum pH and pH stability at pH 3–9 40°C were investigated. pH buffers were acetic acid-sodium acetate solution, PBS solution and Tris-HCl solution at different pH final concentrations of 50 mM.

Definition of unit enzyme activity: The amount of enzyme solution required to hydrolyze dextran to obtain 1 μmol of isomaltose in 1 min is one unit of enzyme activity (U). The formula is as follows:

$$\mathrm{Dextranase}\;\mathrm{enzyme}\;\mathrm{activity}\;\left(U/\mathrm{mL}\right)\;=\frac{\mathrm{Reduced}\;\mathrm{sugar}\;\mathrm{quality}\left(\mathrm{\mu g}\right)\times \mathrm{Dilution}\;\mathrm{times}}{\mathrm{Molecular}\;\mathrm{weight}\;\mathrm{of}\;\mathrm{glucose}\left(g/\mathrm{mol}\right)\times \mathrm{Reaction}\;\mathrm{time}\left(\min\right)\times \mathrm{Enzyme}\;\mathrm{liquid}\;\mathrm{volume}\left(\mathrm{mL}\right)}$$

### Analysis of dextran hydrolysates of SP5 and its variants

The 50 μL enzyme solution was mixed with 1.5 ml of 3% dextran T70 and reacted at 30°C for 240 min; boiled for 5 min; and centrifuged at 8,000 rpm for 5 min to remove the protein precipitate. The supernatant was filtered through a 0.22-μm filter membrane for HPLC analysis. The chromatographic column was waters sugar-park 1, the detector was a differential refractor, the mobile phase was ultrapure water, the flow rate was 0.4 ml/min, and the column temperature was 75°C.

## Results

### Molecular docking and 3D model analysis of mutated protein

**FIGURE 1:**
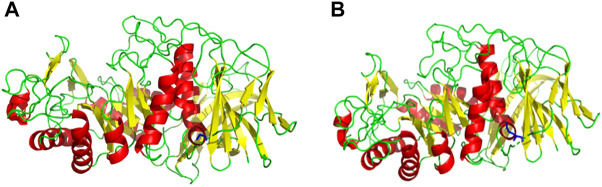
SWISS-MODEL predictive model. **(A)** 3D model of an E454G mutant enzyme. **(B)** 3D model of an unmutated enzyme. Red: α-helix, yellow: β-fold, green: irregularly curled, blue: mutation site.

**FIGURE 2:**
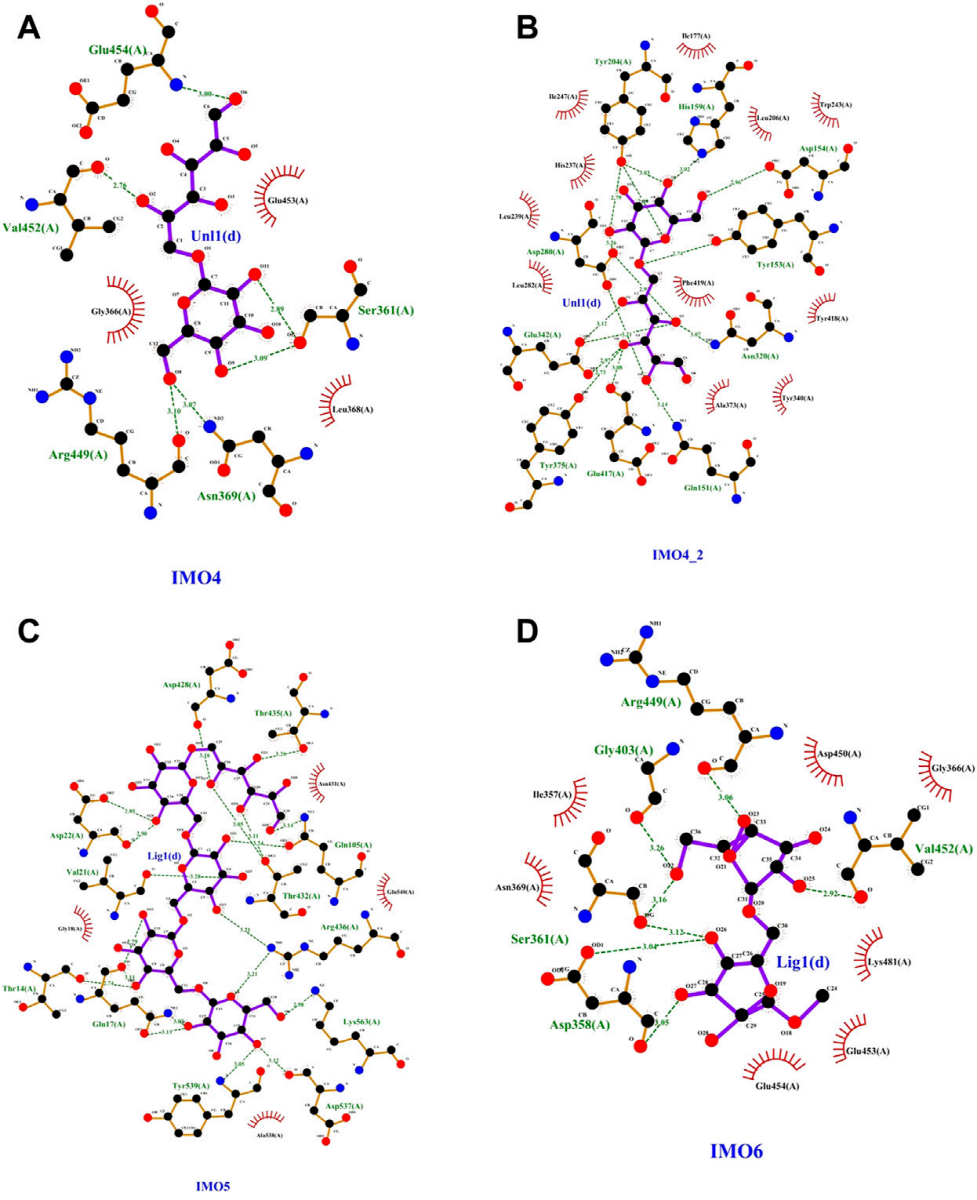
SP5 protein model with **(A)** and **(B)**IMO4, **(C)** IMO5, and **(D)** IMO6 molecular docking results.

Based on the amino acid sequence comparison of SP5 in DANMAN with that of GH66 family hydrolases and 3D modeling on the SWISS-MODEL website, we obtained the SP5 model. SP5 has 26 β-folds, 10 α-helices, and some irregular coiling (Figure 1). Through NCBI prediction, SP5 is known to have a chemical substrate-binding domain (LEU150-LEU430) with a catalytic structural domain (ASP275-ASP345). AutoDuck Vina revealed that GLU454 could successfully dock with both IMO4 and IMO5 with binding energies of −5.4 and −6.9 kcal/mol, respectively, and both could form hydrogen bonds with nitrogen atoms. ASN369 could successfully dock with IMO6 with a binding energy of −10.1 kcal/mol, and the docking of TYR539 with IMO5 was successful with a binding energy of −7.2 kcal/mol. IMO5 could form hydrogen bonds with nitrogen atoms (Figure 2).

### PCR of mutant gene and plasmid recombination

**FIGURE 3:**
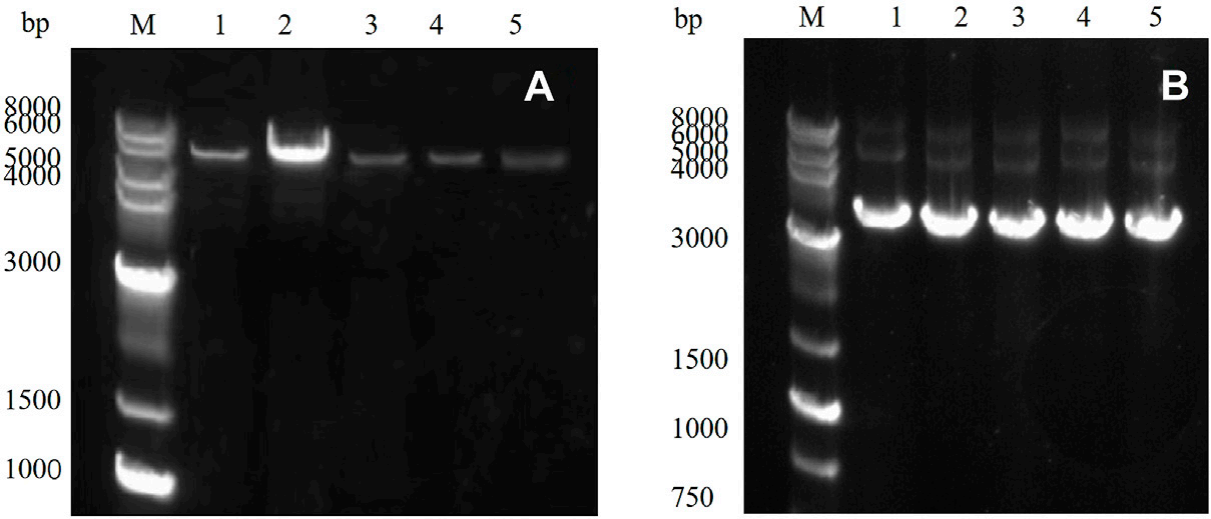
**(A)** PCR product of the target gene: M: marker; Lanes 1–5 represent E454K, E454G, Y539F, N369F, and Y153N, **(B)** *E. coli* DH5α plasmid in receptor cells: M: marker; Lanes 1–5 represent E454K, E454G, Y539F, N369F, and Y153N.

PCR amplification was performed using a rapid sentinel mutation kit based on the predicted five mutation sites, and the results were detected through nucleic acid electrophoresis. As observed in Figure 3A, the mutated gene had a clear band at 5,000–6,000 bp, which was consistent with the predicted results, and the PCR results were accurate.

### Mutagenesis, vector construction, and induced expression of mutated gene

The PCR product was introduced into the *E. coli* DH5α receptor cells and cultured, and the plasmid was extracted. The results were detected through 1% agarose gel electrophoresis (Figure 3B). Obvious bands proved that the target gene had been successfully transferred into the *E. coli* DH5α receptor cells. The plasmid was then transferred into the *E. coli* BL21 (DE3) expression cells to induce expression.

**FIGURE 4:**
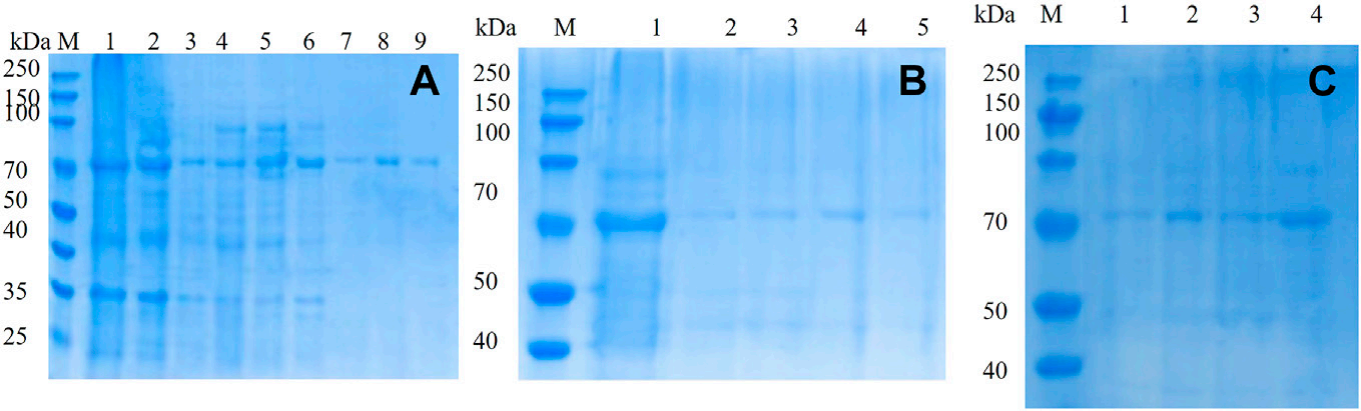
10% SDS-PAGE electrophoresis of mutant **(A)** mutant E454G; M: marker; Lanes 1–9 represent unmutated crude enzyme, E454G mutant crude enzyme and E454G mutant enzyme purified by magnetic bead with the buffer containing 20, 40, 60, 80, 100, 200, and 300 mM imidazole; **(B)** M: marker; Lanes 1–5 represent E454K, E454G, Y539F, N369F, and Y153N; **(C)** M: marker; Lanes from1–4 represent Y539L, Y539W, Y539M, and Y539V.

The mutated dextranase was purified using a nickel column and eluted with 20, 40, 60, 80, 100, 200, and 300 mM imidazole. As shown in Figure 4A, mutant E454G has been successfully expressed. The mutant E454G showed a clear single band near 70 kDa, as the molecular weight of the dextranase is about 66 kDa. Also, the E454K, E454G, Y539F, N369F, and Y153N, Y539L, Y539W, Y539M, and Y539V could been purified (Figures 4B,C). This demonstrated the successful expression of the mutated gene.

### Study of enzymatic properties of mutant enzymes

**FIGURE 5:**
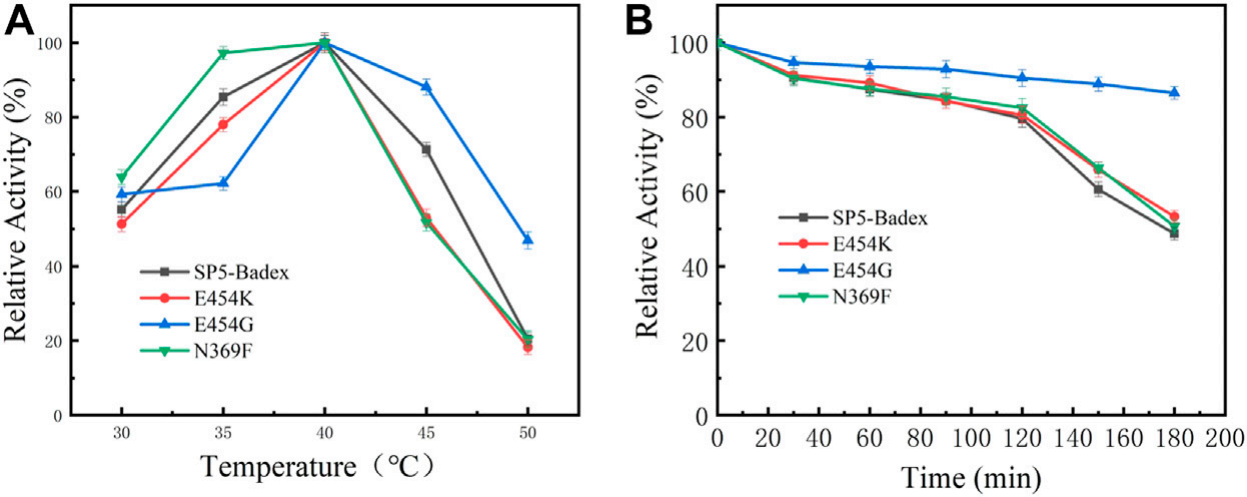
Enzymatic activity of mutant enzymes. **(A)** Optimal activity of temperature, **(B)** Temperature stability of the mutant enzyme at 45°C.

As shown in Table 4 and Figure 5, the optimum temperature of five mutant enzymes were 40°C, however, E454G showed more thermostability. It could maintain 86.5% enzyme activity after 180 min at 45°C, and it increased 37.8% compared with SP5-Badex. E454K and N369F could keep 53.3% and 50.7% of original enzyme activity after 180 min at 45°C, and it still higher than SP5-Badex. Furthermore, the optimum pH of E454K was shifted from 5 to 6, and the pH stability of E454G, E454K, and N369F were changed in broad range. SP5-Badex was stable at pH 5–6; the mutant enzymes were stable at pH 5–7.

**TABLE 4:** Comparison of enzymatic properties and hydrolysates of mutant enzymes.

Enzyme	Specific activity (U/ml)	Optimum temperature (°C)	Thermal stability (°C)^a^	Optimum pH	pH stability^a^	Products
SP5-Badex	0.48	40	≤40	5	5–6	IMO4 52.50%
E454K	0.9	40	≤40	6	5–7	IMO4 56.21%, IMO3 0.53%
E454G	1.96	40	≤45	5	4–7	IMO4 58.12%, IMO3 1.69%, IMO2 0.71%
Y539F	0	0	0	0	0	IMO4 49.13%
N369F	1.2	40	≤40	5	5–7	IMO4 56.67%, IMO3 1.57%, IMO2 0.58%
Y153N	0	0	0	0	0	IMO4 48.16%

a Range at which residual activity was >70%, 0 means no enzyme activity, IMO3 means isomaltotriose, IMO2 means isomaltodioside.

### Study of enzymatic properties of saturated mutant enzymes

**FIGURE 6:**
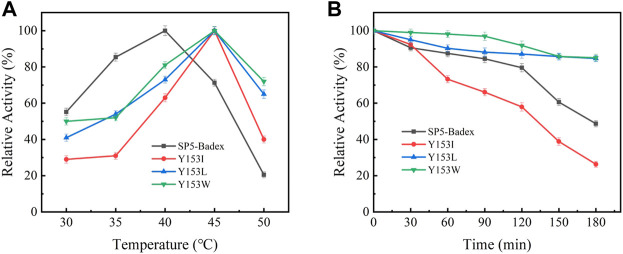
Enzymatic activity of mutant enzymes. **(A)** Optimal temperature for saturation mutation, **(B)** Temperature stability of saturable mutant at 45°C.

**FIGURE 7:**
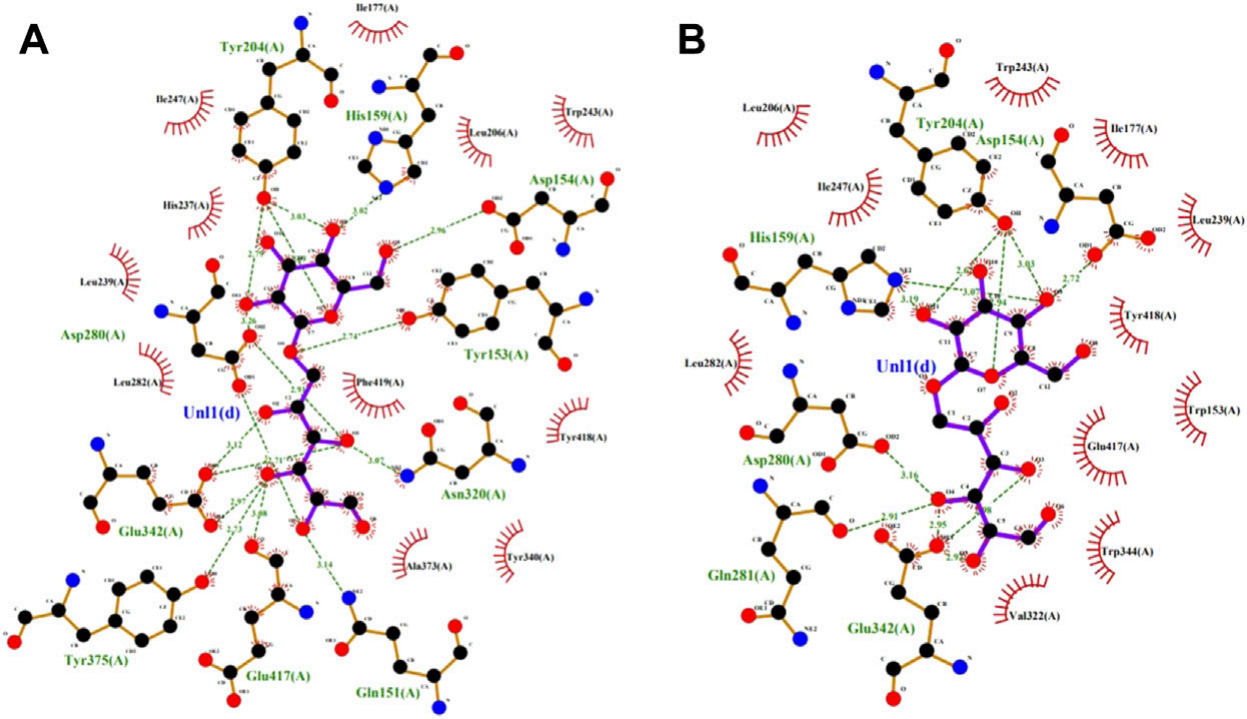
SP5-Badex TYR204 site docking results with IMO4 molecule **(A)**, Y153W mutant TYR204 docking results with IMO4 molecule **(B)**.

The saturation mutation results are presented in Table 5 shows that 15 mutant enzymes had no enzymatic activity, whereas three mutant enzymes exhibited enzymatic activity. Figure 6 shows that the optimum temperature for Y153W’s enzyme activity was increased by 5°C, and the thermal stability for 180 min was also increased to 45°C. At 45°C, enzyme activity of Y153W was increased by approximately 50% compared with that of the unmutated SP5-Badex. At 50°C 30 min, the enzymatic activity of Y153W was retained for 43.02%. The thermal stability of Y153I was significantly lower than that before mutation, probably because of the change in the structure which made Y153I more sensitive to temperature and more suitable for a low-temperature environment. The pH requirement of Y153W shifted to acidic conditions, and the enzyme became more suitable for acidic environments. Y153L retained >70% of its enzyme activity at pH 3–8 for 1 h, indicating that the enzyme was less sensitive to pH changes and more suitable for enzyme production under more acidic or alkaline environments. The enzyme activity of Y153I changed mainly at the optimum temperature and pH, with the optimum temperature reaching 45°C and the optimum pH reaching 6. Thus, Y153I was more suitable for hot and neutral environments. Meanwhile, we re-molecularly docked the Y153W mutant and IMO4 with AutoDock Vina, and the results showed that the mutant changed the substrate binding pocket, hydrogen bonding to hydrophobic interaction at the Y153 site, and analysis of the site binding energy revealed that the binding energy of TYR204 that closed to TRP153 was reduced from the original −6.7 kcal/mol to −7.2 kcal/mol. It might suggest the mutant enzyme was with affinity with substrates (Figure 7).

**TABLE 5:** Enzymatic properties and hydrolysates of 18 saturated mutant enzymes.

Enzyme	Specific activity (U/ml)	Optimum temperature (°C)	Thermal stability (°C)^a^	Optimum pH	pH stability^a^	Products
SP5-Badex	0.89	40	≤40	5	5–6	IMO4 52.5%
Y153H	0	0	0	0	0	IMO4 50.76%
Y153R	0	0	0	0	0	IMO4 60.36%
Y153K	0	0	0	0	0	IMO4 43.1%
Y153I	1.41	45	≤40	6	5–6	IMO4 57.16%, IMO3 1.03%
Y153L	0.91	45	≤45	5	3–8	IMO4 60.07%, IMO3 1.71%
Y153W	2.67	45	≤45	5	4–6	IMO4 64.97%, IMO3 3.07%
Y153A	0	0	0	0	0	IMO4 54.13%
Y153M	0	0	0	0	0	IMO4 45.74%
Y153P	0	0	0	0	0	IMO4 48.32%
Y153C	0	0	0	0	0	IMO4 45.24%
Y153F	0	0	0	0	0	IMO4 50.27%
Y153V	0	0	0	0	0	IMO4 45.03%
Y153G	0	0	0	0	0	IMO4 47.54%
Y153S	0	0	0	0	0	IMO4 49.63%
Y153Q	0	0	0	0	0	IMO4 52.43%
Y153D	0	0	0	0	0	IMO4 50.19%
Y153E	0	0	0	0	0	IMO4 47.69%
Y153T	0	0	0	0	0	IMO4 49.79%

a Range at which residual activity was >70%, 0 means no enzyme activity, IMO3 means isomaltotriose, IMO2 means isomaltodioside.

**FIGURE 8:**
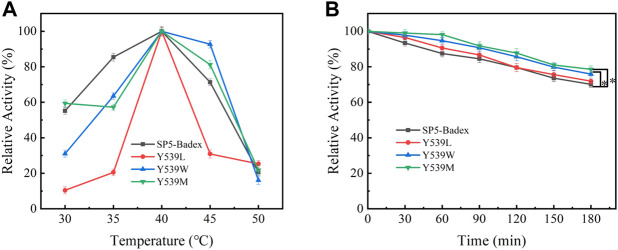
Enzymatic activity of mutant enzymes. **(A)** Optimal temperature for saturation mutation; **(B)** Temperature stability of saturable mutant at 40°C [*significant difference (*p* < 0.05)].

The results of the simultaneous validation experiments showed that three of the four mutant enzymes (Table 6; Figure 8), Y539L, Y539W, and Y539M, had enzyme activities of 0.37 U/ml, 1.32 U/ml, and 1.28 U/ml, respectively. The optimum temperature was 40°C and the optimum pH was 5. After holding at 40°C for 180 min, the enzyme activity of Y539W and Y539M could be retained above 75%, which was significantly higher 5.8% and 8.5% than that before the mutation. The enzyme activity of Y539W could still reach more than 75% after holding at pH 3-6 for 1 h, which was more adapted to the acidic environment and was more consistent with the results of saturation mutation.

**TABLE 6:** Comparison of enzymatic properties and hydrolysates of Y539 mutant enzyme.

Enzyme	Specific activity (U/mL)	Optimum temperature/(°C)	Thermal stability/(°C)^a^	Optimum pH	pH stability^a^	Products
SP5-Badex	0.89	40	≤40	5	5–6	IMO4 52.5%
Y539L	0.37	40	≤40	5	5–6	IMO4 51.08% IMO3 4.2%
Y539W	1.32	40	≤40	5	3–6	IMO4 59.98% IMO3 2.03% IMO2 0.84%
Y539M	1.28	40	≤40	5	5–7	IMO4 62.01% IMO3 2.01% IMO2 1.28%
Y539V	0	0	0	0	0	IMO4 55.03% IMO3 0.24%

a Range at which residual activity was >70%, 0 means no enzyme activity, IMO3 means isomaltotriose, IMO2 means isomaltodioside.

### Mutant enzyme HPLC analysis

**FIGURE 9:**
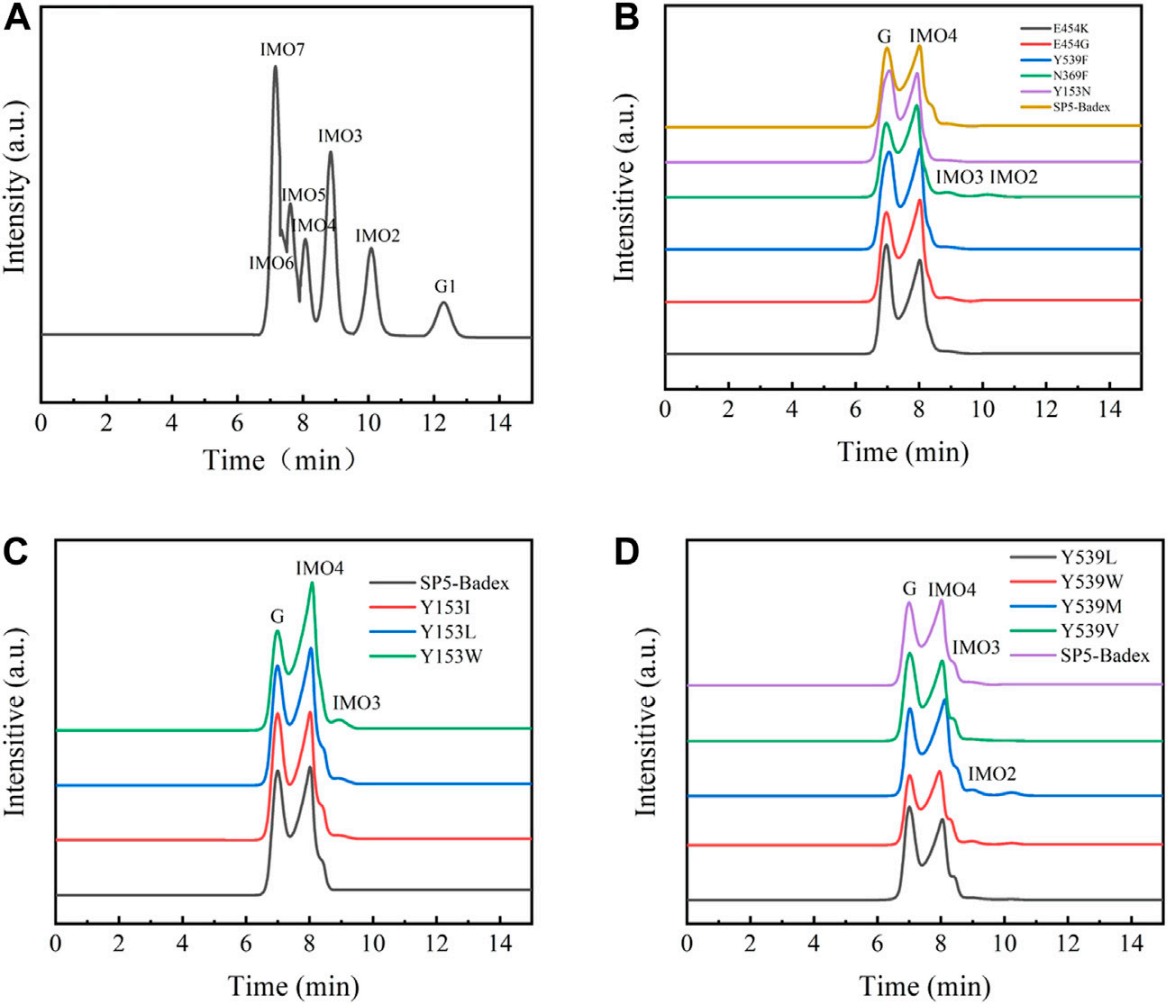
HPLC analysis of mutant hydrolysates (G1: Glucose, G Substrate dextran T70) **(A)** Standard of IMO; **(B)** E454K, E454G, Y539F, N369F, and Y153N mutant enzyme hydrolysates; **(C)** Y153I,Y153L, and Y153W mutant enzyme hydrolysates; **(D)** Y539L, Y539W, Y539M, and Y539V mutant enzyme hydrolysates.

As shown in Figure 9, the hydrolysate of SP5-Badex was IMO4, and after mutation, the hydrolysates of the E454G mutant dextranase increased and included IMO3 and IMO2.

As presented in Table 4, E454K, E454G, and N369F had an increased content of IMO3, IMO3 with IMO2, and IMO3 with IMO2, respectively, compared with SP5-Badex, while the content of IMO4 also increased significantly by 3.71%, 5.62%, and 4.17%, respectively. By contrast, in Table 5 and Table 6, Y539F and Y153N showed a slight decrease in the IMO4 yield. The increase in IMO4 content will lay some foundation for the subsequent prebiotic experiment. According to the saturation mutation results, the IMO4 content of Y153W was 64.97%, which was approximately 12.47% higher than that of SP5-Badex, and was the highest all the mutant dextranase. The IMO4 content of Y153I, Y153R, and Y153L also increased significantly. Similarly, IMO4 yields of Y539W and Y539M were 59.98 % and 62.01%, respectively (Table 6; Figure 8D), which were significantly higher. Tryptophan and methionine are non-polar amino acids, and results are consistent with the speculation of the conclusion of the saturation mutation.

According to previous studies, several factors affect the thermal stability of an enzyme, including electrostatic interactions (Strickler et al., 2006), hydrophobic interactions (Gromiha et al., 2013), hydrogen bonds (Pace, 1995), disulfide bonds (Bader et al., 2018), aromatic ring interactions (Burley and Petsko, 1985), and amino acid composition (Huang et al., 2015). Therefore, we hypothesized that arginine is a basic amino acid; isoleucine, leucine, and tryptophan are nonpolar amino acids, and tyrosine is a polar amino acid and if the amino acid at the position is a nonpolar amino acid, the hydrophobic interactions will change and the protein will rigidly bind and change its conformation, resulting in greater binding and thermal stability of the enzyme. This study provides a solution for the problem of instability of marine origin dextranase in a thermal environment and proposes a scheme to obtain a single IMO4 with higher content, thereby laying a solid foundation for future research on intestinal prebiotics.

## Conclusion and discussion

In summary, we identified mutation sites through molecular docking and successfully targeted mutations to obtain 23 mutant enzymes. A total of six mutant enzymes were detected to exhibit enzyme activities, but all mutant enzymes were found to have hydrolysates after HPLC, which proved that all mutant enzymes completed mutation expression. However, the dextranase activity was measured by DNS method that could measure reduce sugar of the hydrolysates. In the experiments, we assumed no enzyme activity might mean no reduce sugar in the hydrolysates, by contrast, higher DP IMOs might be produced. So, we detected the hydrolysates with the HPLC if the mutant have no enzyme activity. After mutation, the enzyme activity of E454G increased the most compared with that of the unmutated enzyme, reaching 3.08 times, and the yield of the hydrolysate IMO4 increased by 5.62% compared with the SP5-Badex. Thermal stability and hydrolysate analyses revealed that the saturated mutant enzyme Y153W performed better than the other mutant enzymes. It not only exhibited enzyme activity when the optimum temperature reached 45°C but also retained a part of its enzyme activity at 50°C. After holding at 45°C for 180 min, the enzyme activity of Y153W was retained at a high level, with a 50% increase in activity over that before the mutation. Our aims are to obtain higher polymerization degree of IMOs, meanwhile, the reduce sugar could be limited. Y153N showed no enzyme activity which indicated not reduce sugar that could be detected by DNS method. We focused on the prebiotic (IMOs) study, and reduce sugar could be absorbed by body. That is the reason we chose Y153 for the mutation saturation analysis. Our results showed the Y153W could yield 3.07% IMO3 and 64.97% of IMO4. It is greatly improved the DP of IMOs in the hydrolysates.

## Data Availability

The original contributions presented in the study are included in the article/Supplementary Material, further inquiries can be directed to the corresponding authors.
